# Risperidone Versus Risperidone Plus Sodium Valproate for Treatment of Bipolar Disorders: A Randomized, Double-Blind Clinical-Trial

**DOI:** 10.5539/gjhs.v6n6p163

**Published:** 2014-07-29

**Authors:** S. Mohammad Moosavi, Mahshid Ahmadi, Mani B. Monajemi

**Affiliations:** 1 Department of Psychiatry, Mazandaran University of Medical Science. Psychiatry and Behavioral Science Research Center, Sari, Iran; 2 Department of Community Medicine, Medical College, Mazandaran University of Medical Science, Sari, Iran; 3 Department of Clinical Psychology and Mental Health Sciences, University of Tehran, Tehran, Iran

**Keywords:** bipolar disorder, risperidone, sodium valproate

## Abstract

**Objective:**

This study compared the efficacy of risperidone monotherapy with risperidone plus valproate in bipolar I disorder, manic phase. Some studies showed the efficacy of risperidone monotherapy in the treatment of bipolar disorder, so we examined this effectiveness in this clinical-trial study.

**Method:**

This 7-week, randomized, single-blind study included 48 bipolar I inpatients manic phase without psychotic features divided in risperidone group (n = 23) and risperidone plus sodium valproate group (n = 25). According to clinical symptoms, 3 categories: complete remission, partial remission and no remission were mentioned in weekly follow-up. Remission rate compared with survival analysis.

**Results:**

The results showed a significant difference in remission rate between risperidone monotherapy and risperidone plus sodium valproate at the 1^st^, 2^nd^ and the 3^rd^ week (p = 0.012, 0.023, 0.027 respectively), It means the remission rate in risperidone plus valproate group was higher in the first three weeks, but at the end of the seventh week, the difference was not statistically significant. There was no significant difference between the two groups in the development of adverse effects.

**Conclusions:**

Risperidone can be effective and well tolerated in both acute manic episodes of bipolar mood disorders.

## 1. Introduction

First Generation Antipsychotics (FGA) had been used for the treatment of acute mania. Development of extrapyramidal side effects (EPS) and tardive dyskinesia (TD) is the major limitation of using these drugs. Second Generation Antipsychotics (SGA) have been increasingly used for management of acute mania since the year 2000. SGAs are shown to induce less extrapyramidal side effects in comparison with FGA (Perlis et al., 2006). Practice guidelines recommend initiating treatment with either a mood-stabilizing agent or SGA followed by therapy (Hirschfeld et al., 2002; Suppes et al., 2005). Randomized, placebo-controlled trials have demonstrated efficacy of SGA (olanzapine, risperidone, quetiapine, ziprasidone, aripiprazole), for treatment of acute manic phase of bipolar disorders (Tohen et al., 2000; Smulevich et al., 2005). Risperidone (average dose 4.1 mg) produced response rates of 43% in comparison with 24% on placebo (Hirschfeld et al., 2004). In another trial, risperidone with mean dose of 5.6 mg produced response in 73% of patients in comparison with 36% observed in the placebo group (6). In a 12-week, single-blind, placebo-controlled trial for acute mania, risperidone (1 to 6 mg/d) produced a response rate of 48%, similar to the response rate of 47% by haloperidol (2 to 12 mg/d) (Smulevich et al., 2005). Sachs et al. assessed the efficacy and safety of risperidone as an adjunctive agent to mood stabilizers in the treatment of acute mania. In a randomized, double-blind trial including patients with bipolar disorder (manic or mixed phase) who were inadequately responding to mood stabilizer (MS), risperidone, haloperidol or placebo was added. The study demonstrated risperidone plus a mood stabilizer was more effective than a mood stabilizer alone, and as effective as haloperidol plus a mood stabilizer (Sachs et al., 2002). Yatham et al. examined the efficacy of combination of MS with risperidone and showed that risperidone was more effective than placebo when combined with lithium or divalproex in acute mania (Yatham et al., 2003). Bipolar mood disorder is a lifetime disorder that has a recurrence rate of as high as 90%. Most studies about treatment of bipolar disorders have a short duration (for example 3 weeks). Therefore, studies evaluating maintenance phase treatment may better predict efficacy of a drug (Gajwani et al., 2006). We compared the effects of risperidone monotherapy with risperidone plus sodium valproate in a 7-week randomized single blind study for assessment of their tolerability, safety and efficacy in improvement of treatment during bipolar therapy.

## 2. Materials and Methods

The study was a 7-week- randomized double-blind - single centered clinical- trial involving manic patients conducted at Sari Psychiatric hospital (Mazandaran province). The recruitment began in 2012 and ended in 2013. Prior to randomization, eligibility was assessed and medical and psychiatric examinations were completed.

Patients with a diagnosis of bipolar I disorder (manic phase without psychotic features) based on DSM-IV-TR criteria were included. Substance dependency, comorbidity with other psychiatric disorders, general medical diseases (hepatic, kidney, respiratory, etc.), age older than 20 or less than 60 and pregnancy were exclusion criteria. Seventy five patients were selected. Informed consent was obtained from first-degree relatives. Ten of them refused to participate. Blood samples were obtained from all patients for measurement of FBS (Fasting Blood Sugar), CBC (Complete Blood Count), Aminotransferase (Liver Function Test), Serum creatinine, lipid profile and electrolytes. All subjects were examined through urine drug screening. The patients were then randomly divided in two groups (group 1 received risperidone versus group 2 received risperidone plus sodium valproate). Eleven patients discharged by attending and 6 of subjects discharged by family members and all of these subjects were excluded.

Demographic data and the numbers of prior hospitalization were registered. Patients in group 1 (n = 23) received risperidone with a starting dose of 6-8 mg per day in divided dose. Patients in group 2 (n = 25) received sodium valproate 800-1200 mg per day in divided dose plus risperidone with the same dose of group 1. Clonazepam (2-3 mg per day) and trihexyphenidyl (4-6 mg pre day) started in divided dose in both groups. None of subjects received Electro Convulsive Therapy (ECT). All subjects were evaluated on the 7^th^, 14^th^, 21th and 49^th^ days after their admission based on DSM-IVTR clinically. Patients in each group were divided in three response groups based on DSM-IV-TR criteria for bipolar disorder (manic episode without psychotic features):

Full remission: without DSM-IV-TR criteria

Partial remission: one or two criteria

No remission: three or more criteria or no change

At the end, considering a P value of ≤ 0.05 as significant, the data were analyzed by survival tests and Chi-square through SPSS 17.

## 3. Results

There was no significant difference between two groups in baseline characteristics (Table 1). The results showed a significantly more remission rate in the second group at the end of 1^st^, 2^nd^ and the 3^rd^ week (Table 2). Furthermore, at the end of the seventh week, there was no statistically significant difference when comparing the two groups with regard to remission (Table 2).

**Table 1:** Demographic data in risperidone and risperidone plus valproate groups

	Group 1 (Risperidone)	Group 2 (Risperidone+ valproate)
**N**	23	25
**Female/Male**	7/16	7/18
**Age(mean+/- S.D)**	26±1.3	24±1.1
**First time hospitalization**	8	10
**History of prior hospitalization**	15	15

(P > 0.05)

**Table 2:** Comparing the rates of complete remission, partial remission and no remission between two groups at 1^st^, 2^nd^, 3^rd^ and 7^th^ week of treatment

		group 1 (Risperidone) n (%)	group 2 (Risperidone+ valproate) n (%)	P
**Complete Remission**	Week 1	7(30.4%)	10(40.0%)	**0.012**
	Week 2	12(52.2%)	15(60.0%)	**0.023**
	Week 3	20(86.9%)	22(88.0%)	**0.027**
	Week 7	21(91.3%)	23(92.0%)	**0.098**

**Partial Remission**	Week 1	9(39.1%)	9(36.0%)	**0.01**
	Week 2	5(21.7%)	5(20.0%)	**0.018**
	Week 3	0(0.0%)	0(0.0%)	-
	Week 7	2(8.7%)	2(8.7%)	**0.078**

**No Remission**	Week 1	7(30.4%)	6(24.0%)	**0.025**
	Week 2	6(26.1%)	5(20.0%)	**0.021**
	Week 3	3(13.0%)	3(12.0%)	**0.072**
	Week 7	0(0%)	0(0%)	**-**

All patients in both groups has been received prophylactic anticholinergic drugs (trihexyphenidyl) 6 mg daily in divided dose) and benzodiazepine (clonazepam) 2–3 mg daily. Adverse events were reported by 7(30.4%) and 8(32.0%) of patients in risperidone alone and, risperidone plus sodium valproate groups, respectively. In both groups, the most commonly reported adverse effects were: somnolence, tremor, dizziness and constipation. None of the participants were excluded for adverse events. No clinically significant changes in vital signs were noted in both groups. Incidence of adverse events was similar in both groups and there was no significant difference between the two groups in the development of adverse effects.

## 4. Discussion

The results of this study showed a significant difference in remission rate between risperidone monotherapy and risperidone plus sodium valproate at the 1^st^, 2^nd^ and the 3^rd^ week (risperidone plus sodium valproate was superior), but at the end of the seventh week, the difference was not statistically significant. None of the participants were dropped out for adverse events. There was no significant difference between the two groups in the development of adverse effects. Our finding was compatible with Schreiner et al. and Smulevich et al. in demonstrating that risperidone alone can be a tolerable and effective choice in the treatment of bipolar mood disorders (Smulevich et al., 2005; Schreiner, 2006).

Poor adherence to medication during maintenance treatment of bipolar mood disorder is common and exposing patients to the higher risk of relapses, rehospitalization and other negative consequences. Long-acting injectable antipsychotic medications have been used to improve treatment outcome during bipolar maintenance treatment. However, risk of extrapyramidal side effects, tardive dyskinesia, and exacerbation of depressive symptoms are some important limitations to the long-term use of depot FGA in patients with bipolar disorder (18). In contrast, SGA have fewer extrapyramidal side effects and have been better tolerated. Some trials have shown that risperidone was effective and well tolerated in both treatment of acute manic episodes and the maintenance therapy (Smulevich et al., 2005; Schreiner, 2006). Recent data suggest that risperidone can be used effectively either in monotherapy or in combination with a mood stabilizer (Schreiner, 2006). Risperidone long-acting injection (RLAI) is the first long-acting preparation of an SGA introduced into clinical practice (De la Gándara et al., 2009). RLAI have benefits of SGA accompanied with long duration of action and is best for long-term treatment adherence of patients with schizophrenia. De la Gándara et al studied the experiences with injectable long-acting risperidone and have shown that RLAI was well tolerated in the patients with schizophrenia and the overall impression of patients, primary caregivers and relatives to RLAI was positive (De la Gándara et al., 2009).

There are some limitations to this trial. For ethical reasons, patients were permitted to leave the trial. Eleven patients discharged by attending and 6 of subjects discharged by family members before completing the trial and all of these subjects were excluded. Serum levels of applied medication were not measured in these trials, so assessment of treatment adherence was relatively difficult. However all patients were admitted and all drugs have been swallowed under the supervision of a trained nurse.

This study allowed enrollment of only non-psychotic patients and more severely ill manic patients with psychotic features excluded from the study. Generalization is limited because of exclusion of psychotic patients and individuals with psychiatric co-morbidities

This study does not indicate that risperidone is a mood stabilizer, but support the need for additional longer-term studies for better estimation of the effectiveness of different medications and the long-term outcomes in patients with bipolar disorders. Risk factors and drug costs should be considered when prescribing a medication for a patient (Patel et al., 2005).

## Authors’ Contributions

SMM conceived and designed the study, collected the clinical data, interpreted them and helped to draft the manuscript. MA participated in the evaluating and statistical analysis and revised the manuscript. SF re-analyzed the clinical data and revised the manuscript. MM interpreted the clinical data, revised the manuscript and helped to draft the manuscript. All authors read and approved the final manuscript.
